# Comparing rates of agreement between different diagnostic criteria for fetal alcohol spectrum disorder: A systematic review

**DOI:** 10.1111/acer.15492

**Published:** 2024-11-21

**Authors:** Graysen Myers, Michael Burd, Marilyn G. Klug, Svetlana Popova, Larry Burd

**Affiliations:** ^1^ University of North Dakota School of Medicine and Health Sciences Grand Forks North Dakota USA; ^2^ Centre for Addiction and Mental Health University of Toronto Toronto Ontario Canada

**Keywords:** agreement, diagnosis, diagnostic criteria, fetal alcohol spectrum disorder, fetal alcohol syndrome

## Abstract

Diagnostic accuracy is important in systems used to diagnose common disorders such as Fetal Alcohol Spectrum Disorder (FASD). Currently, no comprehensive study has examined rates of agreement between different diagnostic criteria for FASD. This study estimates the likelihood that a diagnosis of FASD using one set of diagnostic criteria will result in the same diagnosis when compared to different diagnostic criteria. A systematic review was conducted to identify articles reporting on the comparison of two or more diagnostic criteria for a diagnosis of FASD. Inclusion criteria required that the study present data that estimated agreement for a diagnosis of FASD or no‐FASD between two or more FASD criteria using two‐by‐two tables or presented data that could be used to generate the tables. Meta‐analyses with confidence intervals were included to demonstrate variability in the estimates. Standardized measures of agreement were assessed using the kappa statistic with 95% confidence intervals and the phi coefficient as a measure of correlation between binary outcomes. The search identified six studies reporting on eight different FASD diagnostic criteria. The studies compared agreement between 17 different pairings of the criteria. For individual children, agreement ranged from 53.7% to 91%. The agreement between the eight different diagnostic criteria ranged from 59.4% to 89.5%. The kappa statistic found that five associations had a kappa ranging from 0.6 to 0.8. This study illustrates that comparisons of multiple pairs of diagnostic criteria are likely to result in considerable variation in diagnoses of FASD for individual children and between different criteria. The lack of agreement between these commonly used systems is likely to affect clinical care and studies where diagnosis is a key variable. Large‐scale multicenter research is needed to examine factors contributing to variation in diagnostic outcomes.

## Graphical abstract

A review of published literature estimating the rate of agreement between eight different criteria for the diagnosis of fetal alcohol spectrum disorder. The agreement between 17 different pairings of these criteria found that agreement on the diagnosis of individual subjects ranged from 53% to 91%. The agreement between the eight different criteria ranged from 59% to 89%. Since many of the criteria are very similar differences between diagnostic centers contributes some variation to rates of diagnostic agreement.

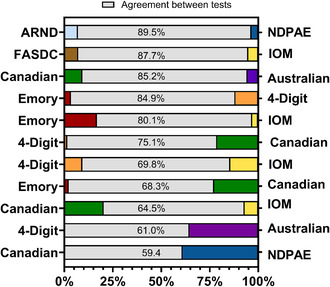

## INTRODUCTION

Prenatal alcohol exposure (PAE) continues to be a pervasive global public health issue. Recent studies have found that 13.5% of pregnancies are alcohol‐exposed, 5.2% were exposed to binge drinking one or more times in the previous 30 days, and 12% had ongoing exposure at the end of pregnancy (Burd, 2020; Gosdin et al., 2022). Alcohol use during pregnancy is an important clinical care issue because it increases the risk for adverse outcomes across the lifespan, including fetal alcohol spectrum disorders (FASD) (Popova et al., 2023). FASD is typically a chronic neurodevelopmental disorder comorbid with over 400 other health conditions (Popova et al., 2016). FASD is also associated with increased development of “secondary conditions,” such as mental health disorders, inappropriate sexual behavior, difficulties with school, increased risk for unemployment, and risk for involvement with juvenile and adult corrections systems (Chudley et al., 2005). Both PAE and FASD increase mortality risk (Thompson et al., 2014).

FASD is a highly prevalent disorder. A study of first‐grade children in the United States. reported a prevalence estimate for FASD of 1.1%–5% (up to one in every 20 children) (May et al., 2018). FASD is more common than many other common birth defects in the United States, such as anencephaly, Down syndrome, spina bifida, and trisomy 18 (Parker et al., 2010). Worldwide, the prevalence of FASD is estimated to be 0.77% or 7.7 per 1000 (Lange et al., 2017).

In addition to the high prevalence and increased rates of comorbidity, people with FASD often require long‐term services from multiple agencies resulting in increased cost of care (Greenmyer et al., 2018). The average annual costs per child with FASD are increased by $22,810 USD and $24,308 for adults (Greenmyer et al., 2018). Healthcare, special education, residential care, encounters with the criminal justice system, and premature mortality all contribute to the high cost of living with FASD. The increased costs and complexity of care also affect caregivers who often incur considerable cost from lost economic opportunity when providing care for people with FASD. Additional productivity losses are attributable to morbidity and premature mortality. Productivity losses leading to reduced income for caregivers of children with FASD are also very likely to have a significant impact on family functioning (Easton et al., 2016; Greenspan et al., 2016). Lastly, caring for people with FASD is associated with additional life stressors and health problems (Oh et al., 2020). Individuals with FASD and their families deserve support in accessing and paying for developmental, health care, and mental health services they need.

FASD is now also considered to be one of the most common causes of developmental disability, including intellectual disability, attention deficit hyperactivity disorder, and learning disabilities (Weyrauch et al., 2017). Unfortunately, the majority of FASD cases are undiagnosed or misdiagnosed (Chasnoff et al., 2015). One common problem contributing to low rates of diagnosis is the lack of standardized screening to identify people at increased risk for FASD (Burd, Klug, & Husark, 2021). This screening deficit can in part be attributed to the lack of a widely accepted FASD screening tool for use by primary healthcare providers in the evaluation of children with suspected PAE and developmental disorders (Waite & Burd, 2023).

Another important factor in the misdiagnosis of FASD globally is the lack of a widely accepted ‘Gold Standard’ diagnostic criterion for FASD. The variability in diagnostic systems for FASD has been discussed previously (Popova et al., 2023). This review discussed the most commonly used diagnostic criteria and demonstrated that the different diagnostic criteria have substantial overlap.

### FASD − diagnostic criteria

The diagnostic criteria currently in use were primarily developed from the studies by Lemoine in 1968 and Jones et al. in 1973, and then a systematic review of available evidence by the Institute of Medicine (Jones et al., 1973, Lemoine et al., 1968, Stratton et al., 1996). Additional refinements have been developed by comparing modifications of these criteria (Chudley et al., 2005; Hemingway et al., 2019; Hoyme et al., 2016). The core variables for the diagnosis of FASD have remained fairly consistent, and typically include documentation of prenatal alcohol exposure, evidence of growth impairment, presence of sentinel facial features, a wide range of structural brain abnormalities and neurobehavioral impairments, and birth defects (Popova et al., 2023). However, the definitions and diagnostic cutoffs for each of these criteria vary depending on the diagnostic criteria utilized (Brown et al., 2019; Burd et al., 2019; Popova et al., 2023).

### Prenatal alcohol exposure

One of the most complex criteria in the diagnosis of FASD is variability and exposure thresholds for PAE or “confirmed PAE”. In the context of exposure assessment, the delineation of the boundaries for confirmed exposure is vague, especially when applied in a clinical setting. Evidence of PAE can be difficult to obtain due to recall bias, stigma for the mother, and inability to contact the mother (due to death, imprisonment, homelessness, or substance use treatment). Limited or no information about PAE in prenatal care records, few or no prenatal care visits, and lack of information in adoption records or for foster care placements add complexity.

While most current diagnostic guidelines require evidence of PAE for a diagnosis, if the complete phenotype for fetal alcohol syndrome (FAS) is present, it may be acceptable to diagnose FAS even if no evidence of PAE is available (Popova et al., 2023). In this case, it is not clear how to proceed if the phenotype for FAS is present, the birth giver denies PAE, and no evidence is available to contradict this report. This could be evidence for categorization as “confirmed no exposure”. In clinical settings, it is not rare that the person who was pregnant may disagree on PAE with other family members who had variable opportunities to observe PAE.

A systematic approach to the collection of exposure data is not widely accepted, and the use of biomarkers for exposure assessment is highly variable. Some delivery hospitals conduct meconium testing using a sample collected from the newborn for the analysis of biomarkers of prenatal drug exposure. A few laboratories do offer an analysis of meconium for alcohol metabolites. However, the laboratory cutoffs for a positive exposure are variable and, in some cases, widely inaccurate (Seputis, n.d.).

Routine screening for PAE utilizing reliable markers such as blood alcohol concentrations are not widely performed (Himes et al., 2015). Models of screening strategies that can be completed during prenatal care, labor and delivery, or immediately after delivery are available and have been reviewed (Dozet et al., 2022; Himes et al., 2015). Recent advances in the use of objective assessments for alcohol use and quantitative measures of PAE have been demonstrated. One example is the use of breathalyzer readings during prenatal care visits (Greenmyer, Klug, et al., 2020). Another quantitative assessment strategy has been testing newborn blood alcohol concentrations. Importantly, blood alcohol concentrations provide objective evidence of maternal, amniotic, and fetal exposure (Hakim et al., 2023; Jung et al., 1980; Schaff et al., 2019). However, blood alcohol concentrations are limited since they assess only recent alcohol use typically only over the course of hours.

Additional complexity in the diagnosis of FASD is related to potential confounding from prenatal polysubstance use (e.g., opioids, tobacco, cannabis, etc.). Prenatal polysubstance is very common among children who are being evaluated for FASD. Biomarkers of PAE are needed to replace the current reliance on maternal self‐report.

### Growth impairment

Evidence of growth impairment (height and weight) is a common criterion for diagnosis of FAS and pFAS but is not always required. The most common cutoffs are values at or below the first, fifth, or 10th percentile. However, considerable variability exists across norms used to assess growth standards for different racial and ethnic populations. It appears to be increasingly accepted that most people with FASD globally do not have growth deficiencies.

### Facial features

Three common sentinel facial abnormalities (decreased palpebral fissure length, thin upper lip, and abnormalities of the philtrum) are commonly assessed, but the number of the abnormalities required for a diagnosis is variable (Hoyme et al., 2016; Popova et al., 2023). The assessment of these important facial features has been partially standardized by the use of lip‐philtrum guides usually presented as a Likert scale from 1 to 5. However, versions of the scales are not available for many racial and ethnic populations, or for different ages (newborns, children, adults, and the elderly). Importantly, most people with FASD do not have characteristic facial features, and even where these features are present, they may fade over time (Jacobson et al., 2021). The presence of facial features is required for a diagnosis of FASD or for diagnosis of some of the categorical diagnoses of FASD depending on the diagnostic criteria being utilized.

### Brain dysfunction

After collecting evidence of PAE, the diagnosis of FASD is most dependent on evidence of brain dysfunction. The strategies for assessment of brain dysfunction in current use are primarily developed for school‐aged children. A single widely accepted set of neuropsychological tests for the assessment of various domains of brain dysfunction is not available. The number of diagnostic tools and strategies utilized to assess brain impairment is very large. In one study from Alberta Canada, the authors found that 173 different assessments were utilized in the assessment of FASD (Coons‐Harding et al., 2019). Among these are different measures for the assessment of developmental achievement in speech and language, fine and gross motor skills, executive function, attention, memory, and multiple other deficits. The use of these tools across multiple racial and ethnic populations is also complex. Another common issue is the complexity of assessment of newborns, infants, and children, many of whom have highly variable comorbid disorders (severe vision or hearing loss, autism spectrum disorders, nonverbal children, or children with intellectual disability).

### Postnatal exposures

The detection and assessment of postnatal exposures among people being evaluated for FASD can be difficult. Many people have had multiple foster home placements for abuse and neglect. The assessment of these adverse childhood experiences (ACEs) as moderators of PAE and FASD severity is highly variable and complex (Kambeitz et al., 2019). In some circumstances, ACEs may be exclusionary, while in some centers they may serve as an index of severity (Kautz‐Turnbull et al., 2023; Price et al., 2017; Tan et al., 2022). In our center, the record for the number of foster care placements has been 31 for a child who was not yet 14 years of age.

### Age at assessment

The age of the person being evaluated is a concern since tools for assessment of the FASD for the very young (ages birth to three) and for adults (ages 21 and up) are limited. By far the largest number of people with FASD would be in the adult, middle‐aged, and elderly populations. The current criteria for diagnosis of FASD are simply not adequate for the assessment of the majority of people in these age groups. This is concerning since in the United States alone, the number of adults and elderly with FASD is likely to be in the millions.

### Diagnostic capacity

While the concept of a multidisciplinary approach for the diagnosis of FASD is widely endorsed, this approach cannot meet the diagnostic capacity demands for a lifelong condition with a prevalence rate of 1%–5% of school‐age children (Dugas et al., 2022; Popova et al., 2020). In addition, few FASD diagnostic clinics are able to assess people of all ages. A recent study demonstrated that huge increases in capacity are going to be required just to have capacity to diagnose the cases in current birth cohorts (Popova et al., 2024). This study demonstrated that the diagnostic capacity needs to be increased by over 60‐fold to offer assessments to people with FASD in much of Canada.

### Benefits of early diagnosis

The early diagnosis of FASD may facilitate access to diagnosis‐informed services for people with FASD (Bisgard et al., 2010). Early recognition may also help identify other family members (e.g., siblings) who may be at risk for FASD. An often underappreciated benefit of receiving a diagnosis of FASD is the potential to support the birth parent to access services to eliminate alcohol use in future pregnancies and prevent exposure (Greenmyer, Popova, et al., 2020). If successful, this could decrease the risk of FASD in younger siblings from future pregnancies. Another is the potential to reduce the risk for the development of “secondary conditions” such as mental health diagnoses, inappropriate sexual behavior, difficulty with school experiences, increased risk for unemployment, and involvement with juvenile and adult justice systems (Chudley et al., 2005).

High prevalence rates, increased rates of comorbidity, high cost of care, the importance of early diagnosis, and the use of multiple sets of diagnostic criteria for FASD demonstrate the need for a systematic review to compare the outcomes between the different criteria. One important feature to consider when comparing these systems would be the rates of agreement for the diagnosis. To date, very few studies have examined this issue. Published studies have generally utilized a strategy where the diagnosis for a cohort of patients evaluated under one set of criteria is compared with the diagnosis when a different set of diagnostic criteria is applied to the same cohort. The rates of agreement between the two sets of criteria are then reported. One report involved a comparison of the 4‐digit code diagnostic assessment and the Institute of Medicine (IOM) modified by Hoyme and colleagues (Hemingway et al., 2019). Another study compared the criteria for ARND and the criteria for NDPAE presented in the Diagnostic and Statistical Manual of Mental Disorders (DSM‐5) (Johnson et al., 2018). A similar study has been reported (Sanders et al., 2017). A study comparing the criteria from the Emory clinic, the 4‐digit code, the IOM criteria and the criteria recommended by the CDC has also been published (Coles et al., 2016). These studies have reported widely divergent results when estimating rates of agreement between the different diagnostic criteria.

The current study utilizes a systematic review and meta‐analysis to estimate the likelihood that a diagnosis of FASD using one set of diagnostic criteria will result in the same diagnosis when compared with a different set of diagnostic criteria. This methodology offers a robust methodology for comparison of multiple studies of diagnostic criteria when compared to different criteria applied to a single patient cohort.

## METHODS

**FIGURE 1:**
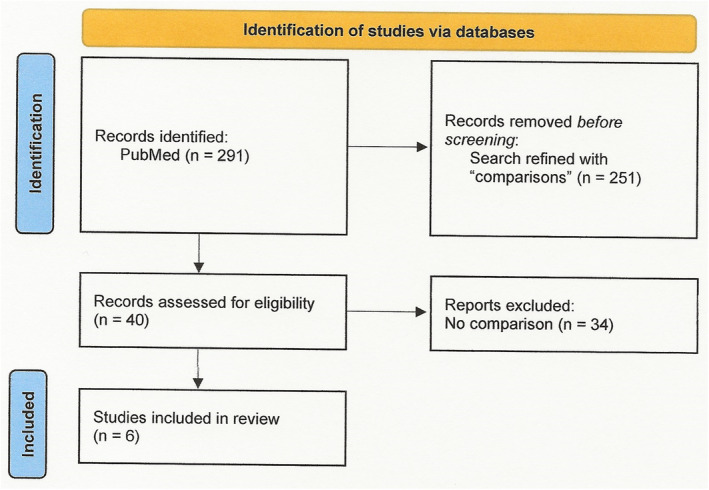
PRISMA diagram of the literature search and individual manuscript review process.

In this study, we sought to identify published papers comparing diagnostic schema for FASD. The initial search in the PubMed database, utilizing the search terms (NDPAE[tiab] OR IOM[tiab] OR Canada*[tiab] OR Australia*[tiab] OR FASDC[tiab] OR ARND[tiab] OR Emory[tiab] OR “4‐digit”[tiab] OR (diagnos*[Title] AND compar*[tiab])) AND (“diagnostic techniques and procedures”[MeSH Terms] OR (diagnos*[tiab] AND alcohol*[tiab])) AND (“fetal alcohol spectrum disorders”[MeSH Terms] OR “fetal alcohol”[tiab]) produced 291 papers (Figure 1 PRISMA diagram). This search includes other categorical diagnostic groups for FASD including partial FASD and FAS. Table S1 contains the individual diagnostic criteria and the categorical diagnoses used in this study. Criteria were then further refined by adding “comparisons” which resulted in a list of 40 studies. The inclusion of terms such as Scottish/UK and CDC did not identify other diagnostic systems that meet our study criteria (Figure 1).

Each of these studies was screened first using the title and abstract, and then where necessary the full‐text level by two of the authors. The reference list for each screened study was also examined to identify additional studies that could be included. The search was completed in July of 2023 with no restrictions on language or geographic region.

The inclusion criteria were: (1) Studies were required to describe the utilization of a well‐described set diagnostic criteria for FASD and a comparison with another FASD diagnostic criteria; (2) Each study had to present data that estimated agreement for the diagnosis of FASD or no‐FASD between the different FASD diagnostic criteria using two‐by‐two tables, or present data that could be used to generate the tables. The exclusion criteria were as follows: (1) Studies which did not report on the comparison of at least two sets of criteria; or (2) Studies where data to generate the two‐by‐two tables was not available.

For each included study, a two‐by‐two table was developed to compare the two sets of diagnostic criteria for agreement and was used for analyses. If not all cells in the table were explicitly defined by the author, they were reconstructed using row or column totals/percents or kappa statistics. For each paper, one set of diagnostic criteria (standard) was selected to be the standard that another set of diagnostic criteria (comparison) would be compared to. The comparison was whether the different diagnostic criteria agreed on the diagnosis of FASD or no‐FASD. The diagnostic criteria (standard) are the columns and the diagnostic criteria being (compared) are the rows in the two‐by‐two tables.

From these tables, the percent agreement (total number agreed positive or negative divided by total number measured), Kappa statistic (a measure of overall agreement or reliability between comparison and standard controlling for chance), and Phi coefficient (a binary correlation between the comparison and the standard) were calculated as measures of overall agreement. The percentage of disagreement was also calculated, for the percentage of people diagnosed as FASD by one criterion, but where the comparison criteria disagreed on the FASD diagnosis. PTC MathCad Prime 7.0 was used to recreate the tables and estimate these statistics. Since this was a study of percent agreement, further assessments like funnel plots, tests for bias, and tau or chi‐square tests for homogeneity were not produced.

Confidence intervals for the percent agreement between measures and the Kappa statistic were calculated using standard error of proportions and the z statistic. Where a standard measure had agreement tested with at least two comparisons, we calculated a composite measure of agreement including confidence intervals with variance adjusted for the sample sizes and variations within each agreement. This provided an estimate of the likelihood of any comparison measure diagnosing FASD when the standard measure diagnosed FASD. General linear mixed models using log–log linkage with proc NLMixed in SAS 9.4 were used to estimate the composite agreements. The log–log linkage is asymmetrical and is a better fit for unbalanced data. The potential for bias in the various studies was primarily controlled with each study. However, in our comparisons we included meta‐analyses with confidence intervals to demonstrate variability in the estimates between the different criteria.

## RESULTS

Six studies were included in this review, reporting on rates of agreement for the diagnosis of FASD or no‐FASD between 17 different pairings of eight diagnostic strategies for FASD (Table 1). For convenience and consistency of citation, the papers published by Hoyme et al. will be referenced as modifications of Institute of Medicine criteria (IOM) (Hoyme et al., 2016). The paper published by Sanders et al. will be referenced as modifications of Canadian criteria (Sanders et al., 2017). For the analysis, one FASD diagnostic criteria was selected as the standard in the columns for the two‐by‐two table, and the other diagnostic criteria was considered the comparison and used in the rows of the table. Three pairs of diagnostic criteria were measured, with each pair combined by addition of cells into a single two‐by‐two table for the analyses.

**TABLE 1:** Measures of agreement between differing sets of FASD diagnostic criteria.

References	Standard	Comparison	*N*	% agree	% comp. Yes stan. no	% Stan. Yes comp. no	Kappa	95% CI	Phi
Hemingway et al. (2019)	4‐Digit	IOM	1392	57.9	2.9	39.1	0.216	0.167 to 0.265	0.292
Coles et al. (2016)			1581	77.4	22.3	0.3	0.570	0.523 to 0.617	0.627
Coles et al. (2023)			2158	87.3	3.9	8.8	0.403	0.322 to 0.484	0.414
Combined			5131	76.3	9.3	14.4	0.492	0.463 to 0.521	0.495
Hemingway et al. (2019)	4‐Digit	Canadian	1392	54.0	3.4	42.6	0.171	0.124 to 0.218	0.242
Coles et al. (2016)			1581	83.7	2.0	14.3	0.630	0.574 to 0.686	0.655
Coles et al. (2023)			2320	87.0	0.2	12.8	0.109	0.011 to 0.208	0.219
Combined			5293	77.4	1.5	21.1	0.470	0.439 to 0.501	0.529
Hemingway et al. (2019)	Canadian	IOM	1392	53.7	21.6	24.7	0.053	0 to 0.107	0.053
Coles et al. (2016)			1581	64.5	35.0	0.6	0.350	0.305 to 0.395	0.451
Coles et al. (2023)			2158	91.0	8.7	0.3	0.159	0.037 to 0.281	0.259
Combined			5131	72.7	20.3	7.0	0.321	0.287 to 0.354	0.340
Hemingway et al. (2019)	Canadian	Australian	1392	85.2	9.3	5.5	0.698	0.645 to 0.752	0.700
Hemingway et al. (2019)	4‐Digit	Australian	1392	64.5	0	35.5	0.333	0.284 to 0.382	0.447
Sanders et al. (2017)	Canadian	NDPAE	82	61.0	0	39.0	0.320	0.133 to 0.506	0.436
Burd et al. (2003)	FASDC	IOM	390	87.7	7.2	5.1	0.656	0.523 to 0.789	0.657
Johnson et al. (2018)	ARND	NDPAE	86	89.5	7.0	3.5	0.730	0.464 to 0.996	0.733
Coles et al. (2016)	Emory	4‐Digit	1581	84.9	3.4	11.7	0.690	0.640 to 0.740	0.701
Coles et al. (2016)	Emory	Canadian	1581	75.1	2.2	22.8	0.480	0.429 to 0.531	0.532
Coles et al. (2016)	Emory	IOM	1581	80.1	16.8	3.0	0.610	0.561 to 0.659	0.633

**FIGURE 2:**
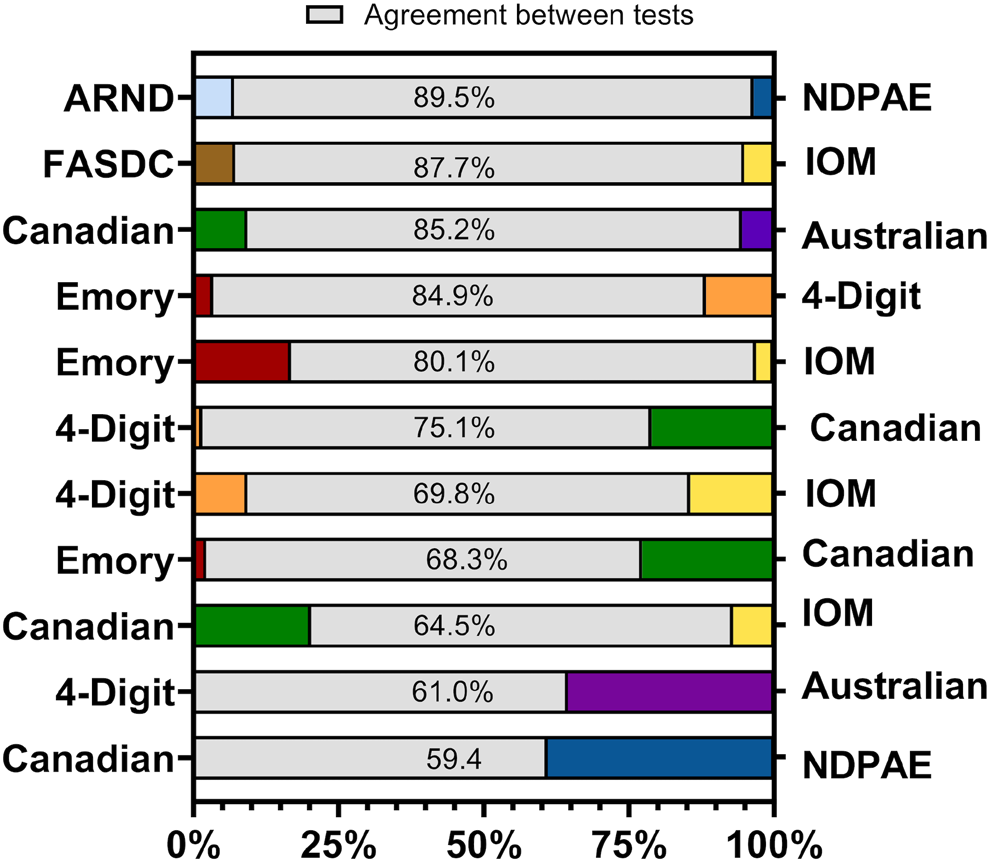
Percent of agreement and disagreement between different diagnostic criteria for FASD. Percent agreement in the center represents the percent of children where both sets of diagnostic criteria have the same diagnosis. The colors on the left and right represent the percent of children who had different diagnoses for each set of criteria.

Table 1 shows the different agreement measures for a diagnosis of FASD estimated by the two‐by‐two tables for available pairs of FASD diagnostic criteria. For each diagnostic criteria pairing, the percent of the total number of children evaluated who had a diagnosis of FASD or non‐FASD from both sets of diagnostic criteria was the percent agreement. The percentage of disagreement was measured in two different ways (Table 1). One method of disagreement is the percentage of children who were defined as non‐FASD by the standard measures but defined as having FASD by the comparison measure (cell B in the two‐by‐two table). A second possible disagreement is when the standard indicated FASD, but the comparison measure said it was no‐FASD (cell C in the two‐by‐two table). The bar graph (Figure 2) describes these agreement and disagreement percentages. Differences in the rates of agreement for categorical diagnosis of FASD relied on reports in the original studies.

The percent agreement between the different sets of diagnostic criteria ranged from 59.4% to 89.5% (Figure 2). Five pairs of diagnostic criteria agreed less than 70% of the time and five pairs agreed over 80% of the time: (Alcohol‐Related Neurodevelopmental Disorder (ARND) and Neurodevelopmental Disorder Associated with Prenatal Alcohol Exposure (NDPAE) 89%, Fetal Alcohol Syndrome Diagnostic Checklist (FASDC) and Hoyme et al. (2016) 88%, Canadian criteria and Australian criteria 85%, Emory criteria and 4‐Digit code 85%, and Emory and Hoyme et al. (2016) 80%).

**FIGURE 3:**
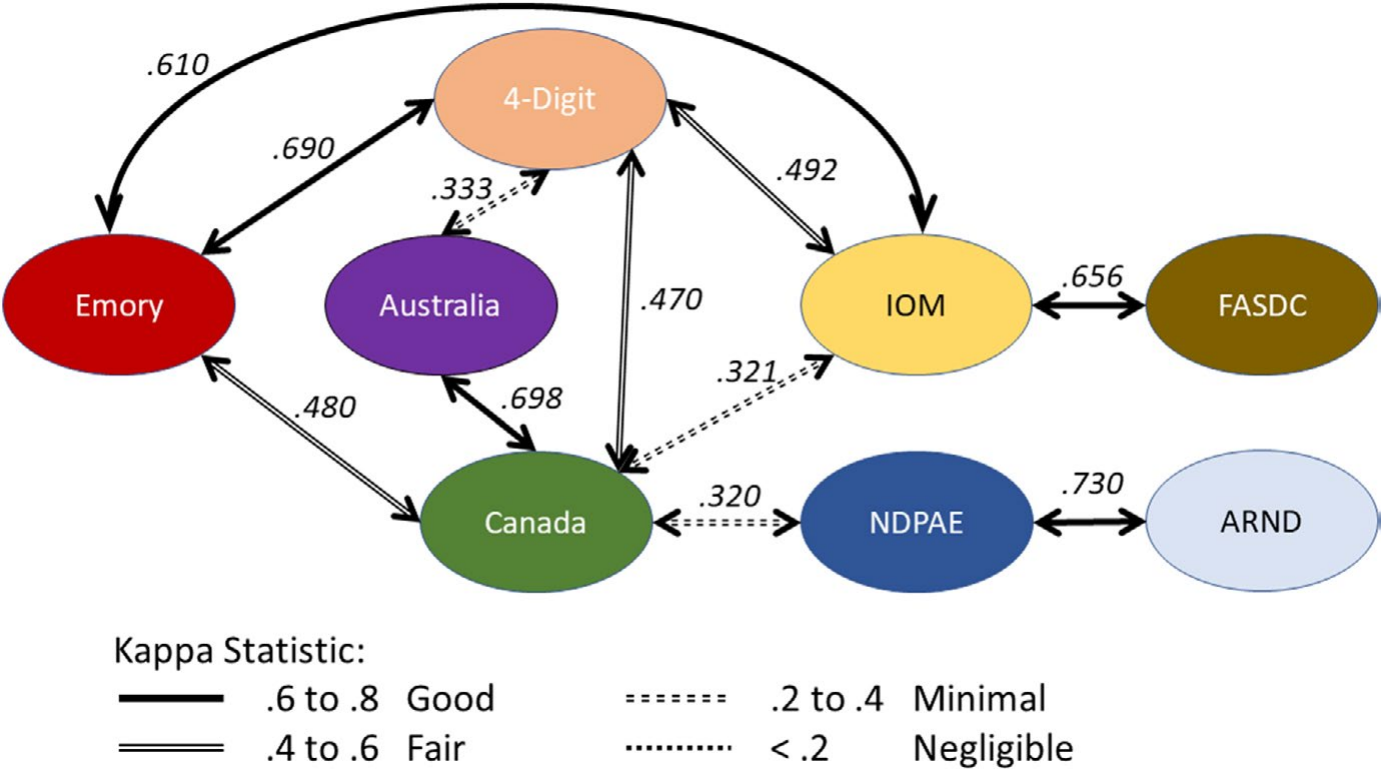
Kappa measures of agreement between different FASD diagnostic criteria.

Figure 3 presents the comparison data for the 17 pairs of criteria. The Canadian criteria were compared five times with other diagnostic criteria and the percent agreement ranged from 59.4% to 82.5%. The criteria from Hoyme et al. for the IOM were compared four times and percent agreement ranged from 64.5% to 87.7%. The 4‐Digit Code was compared four times and the percent agreement ranged from 61.0% to 84.9%. The Emory Criteria were compared three times, and the percent agreement ranged from 63.8% to 84.9%.

Disagreement on the diagnosis between pairings of criteria are presented in two ways. The colored bars in Figure 2 indicate the criteria listed on that side is more likely to disagree with the FASD diagnoses when compared to the criteria listed on other side. A larger colored bar represents a diagnostic criterion resulting in increased disagreement in the diagnosis when the criteria referenced on the other side of the figure diagnosed FASD. As an example, the fourth bar from the top in Figure 2 demonstrates that the 4‐Digit Code (right side) was more likely to indicate a different diagnosis for FASD when compared with the Emory criteria (left side). Similarly in the fifth row, the Emory criteria (left side) was more likely to disagree on the diagnosis of FASD when compared with the IOM criteria (right side). The Australian criteria had a larger percentage (35%) of children whose FASD diagnosis disagreed with the 4‐Digit code criteria. The NDPAE criteria also had a large percentage (39%) of children with a different FASD diagnosis when compared to the Canadian criteria.

Table 1 also shows standardized measures of agreement (kappa statistic) with 95% confidence intervals and the phi coefficient as a correlation measure between binary outcomes. All the kappa statistics had confidence intervals greater than 0 except for one Canadian and IOM association. The kappa statistics were plotted together with the linkage between tests in Figure 3. Five of the associations were in good agreement with kappa statistics between 0.6 and 0.8. Three were fair and two were minimal. The strongest multiple linkage appears to be between the 4‐Digit, Emory, and the IOM.

**FIGURE 4:**
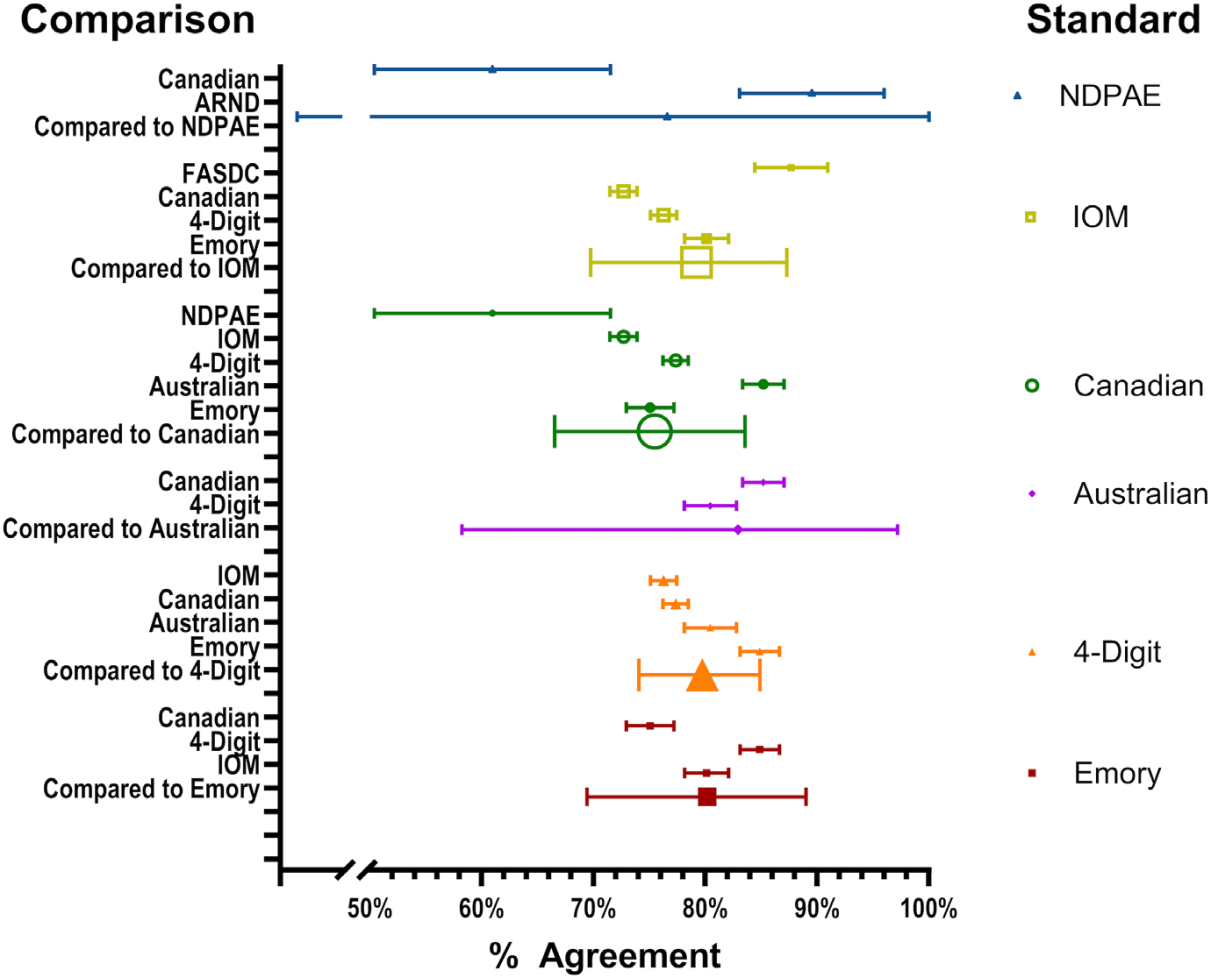
Percent agreement for FASD diagnostic criteria compared with multiple other sets of criteria combined. Small shapes (i.e., square, circle, triangle, and colored square) represent the individual percent agreement for each set of diagnostic criteria, as seen on the right side of the figure with the comparison criteria. Large shapes represent the comparison of the standard criteria to the combination of the comparison criteria.

The Canadian criteria had five comparisons, the IOM and 4‐Digit were each compared with four other tests, the Emory criteria was compared with three others, and the Australian and NDPAE criteria were compared with two others (Figure 4). To estimate how well one set of criteria agreed with the diagnosis from any of the other comparisons, an overall agreement percentage was calculated using log–log linkage in a non‐linear mixed model, a meta‐analyses strategy that controls for non‐normality in the distribution of the percentages. Figure 4 shows the forest plot of the original agreement percentages and the agreement percentage combined.

The confidence intervals on the individual agreements are not adjusted. The NDPAE and the ARND pairs had 89% agreement and the NDPAE and Canadian criteria had 61% agreement. When combined, the agreement was 77% but the confidence interval was very large due to the small number of children included in the studies. The IOM was compared to four other criteria, two with high agreement and two with low. The combined agreement rate was 79%. A child receiving a diagnosis from the IOM criteria has a 1 in 4 chance of receiving a different diagnosis when the FASDC, Canadian, 4‐digit, or Emory criteria are used. Agreements of one test with multiple others ranged from 75% (Canadian criteria) to 83% (Australian criteria).

## DISCUSSION

In this study, we found that eight different FASD diagnostic criteria agree on the diagnosis of FASD or no‐FASD for an individual child in 53.7% to 91% of the cases (Table 1). When the individual diagnostic criteria were compared, the percent agreement was similar, ranging from 59.4% to 89.5%. The kappa statistic, a measure of overall agreement or reliability between the diagnostic criteria (standard and comparison criterion) demonstrated that five associations had a kappa score ranging from 0.6 to 0.8, indicating good agreement.

The variability in diagnosis was surprising since the different criteria all have extensive overlap due to the close interdependence between these criteria as the different approaches were developed and modified over time. As we noted above, modifications of the criteria have often included comparisons of the criteria as a validation of face validity.

Diagnostic accuracy is important in medicine. Disagreement in diagnostic outcomes between different criteria may impact people's confidence in identifying and caring for people with FASD. For example, children who are receiving services in one community may not be eligible for services when children and families move to another community if differing diagnostic criteria produce different outcomes. For children without an accurate diagnosis, the behavioral dysregulation that often accompanies FASD can increase the likelihood that patients will be misdiagnosed. This could increase the likelihood of inappropriate treatment. To the extent that a diagnosis of FASD is meaningful and that some of the services needed are diagnosis‐informed, the inability to access these services may be harmful.

### Limitations

While this study has several strengths, including results from several diagnostic centers and multiple different diagnostic criteria, it also has several limitations.

- The available data, different referral patterns, age limitations, and differing exclusionary criteria for access to the reporting clinics limit the comparisons that can be made.
- The number of comparisons that could be examined for each set of criteria was modest. As an example, it was not possible to stratify the comparisons by age. The impact of differing ages may be important if some of the criteria are better suited to specific age groups.
- The impact of phenotype severity could not be assessed between pairs of diagnostic tests.
- Variation in the reliance on, and the prevalence of, FASD facial features between centers may bias results.
- The impact of differing patterns and methodology for assessment of comorbidity could not be examined.
- Data were not available to examine either the cost or burden of the differing diagnostic systems. These factors may impact referral patterns and affect which patients attended at the individual clinics.
- It was not possible to compare exposure detection, assessment strategies, or dosimetry of exposure. This issue may be especially concerning where exposure status is used as an initial screen for patients to access the diagnostic clinic. Ideally, having one widely accepted set of exposure criteria that has a low burden for implementation, adequate epidemiologic performance criteria, low cost, and ease of interpretation would be very useful.
- The agreement between criteria used to diagnosis FASD is highly variable, with significant variation for both individual children and the different diagnostic criteria. We could not determine where the disagreement between cases or individual diagnostic criteria occurred.
- It was not possible to determine which set of diagnostic criteria may be the most appropriate or represent a “Gold Standard.”
- The phenotype of FASD may be much broader than previously described. This may account for some of the variability in outcomes found in this study. Recent prevalence data suggests that most cases of FASD are not FAS but rather other manifestations of FASD (Popova et al., 2023).
- The phenotype for FASD may be both age and development dependent. Children with FASD may have considerable variation in the developmental manifestation of comorbidity, which is important in the diagnosis of FASD (Burd, 2016; Burd & Popova, 2019). Many of the comorbid disorders accompanying FASD are difficult to diagnose early in life (speech and language disorders, intellectual disability, depression, anxiety, substance use or potential for criminal activity). In most cases, FASD becomes more complex as the child ages into adult life (Burd, 2016). As we have previously reported, comorbidity often determines complexity in FASD, especially for people who do not have severe birth defects (Burd, Klug, O'Connell, et al., 2021). The presence of severe intellectual disability, blindness or visual impairment, and deafness or hearing loss are examples of this effect.
- Variation in phenotype could also be influenced by duration and timing of exposure, dosimetry of exposure, exposure to ACEs, number of comorbidities, and age at the time of assessment.

### Future research

The data presented in this study were primarily retrospective comparisons made after the diagnosis of FASD had been completed. Planning for additional research in this area might consider development of a prospective multicenter trial. A prospective trial examining the performance of multiple sets of diagnostic criteria used in the diagnosis of FASD may be much more variable. This possibility would need to be carefully considered in the power analysis to determine sample size, the number of diagnostic centers to be included, and the number of diagnostic criteria that could be compared.

A trial that featured standardized patient selection criteria would be very useful. Crucial variables would include age group, comorbidity status, confidence in exposure assessment detection, and dosimetry of exposure. The trial would optimally include some patients with other developmental disorders and genetic disorders. A trial featuring random assignment of these patients between diagnostic centers would be very useful. The centers would then utilize a predetermined data collection protocol that would include the essential variables for each of the different diagnostic criteria. This would be used as the individual assessment of each study subject.

If these design elements are not possible, an alternative option might feature a data collection phase followed by an analytic phase where the final diagnosis for each subject is derived from the centralized project dataset. While this design may resemble many of the smaller studies already included in this review, the strategy would have advantages. The participating centers could develop agreement or standardization of the evaluation components prior to the launch of a multicenter trial. For example, how can the exposure assessment be structured so that multiple FASD diagnostic criteria can be included in a single multi‐center trial? These design features would allow for a more accurate comparison of the epidemiologic performance criteria for the multiple diagnostic criteria. If the majority of children diagnosed with FASD do not have abnormal facial features, it would be important to assign weights to ensure that the presence of characteristic facial features would not become a source of significant confounding when the algorithms used to determine diagnostic status are utilized. Perhaps one of the most important improvements would be the opportunity to randomly assign individual subjects to different diagnostic criteria.

The quality and efficiency of a multicenter trial might be greatly improved if a telehealth component were included, since this would allow random assignment of subjects to different diagnostic criteria applied at different diagnostic centers. This would also greatly enhance the study of differing neuropsychological assessments to estimate brain dysfunction and impairment.

Several other considerations for the design of such a trial should be considered. It will be important to ensure the trial design will develop data that can be used to examine the benefits and risks from a harmonized or standardized set of diagnostic criteria. The study should allow for the possibility that FASD has a very broad phenotype that is substantially dependent on age, the developmental trajectory of the individual child, and the manifestation of their impairments from comorbidity. It may not be possible or even desirable to develop a single, comprehensive diagnostic protocol for use across different states/countries, rural versus urban locations, and differing health care systems to be used in the diagnosis of people over a 70–80‐year age span with differing phenotype severity, severity of comorbidity, and number of comorbid conditions for individuals.

The diagnosis of FASD in newborns, infants, toddlers and preschoolers is likely far more difficult than for older age groups, and levels of disagreement may be much greater than found in this study. However, the importance of early diagnosis as a strategy to facilitate entry into diagnosis‐informed interventions is likely to be very significant. The overwhelming majority of people with FASD are adults and the elderly, who are likely to require a very different approach to diagnosis, especially when compared to the strategies used for school‐age children.

Future efforts to increase access to FASD diagnostic centers are urgently needed. We need to be able to find people with FASD to be able to treat them. For the millions of people with the disorder and their families who care for them, it is time for a change. FASD is a global public health problem, so one important outcome for future studies will be to develop diagnostic criteria that can be applied globally and improve access for adults and the elderly. Let us commit to reconceptualize FASD from a childhood problem to a lifespan disorder of global significance.

## CONFLICT OF INTEREST STATEMENT

All authors declare no conflicts of interest.

## Supporting information


Table S1.

## Data Availability

The data that support the findings of this study are available from the corresponding author upon reasonable request.
