# Supplementary material for: Comparing rates of agreement between different diagnostic criteria for fetal alcohol spectrum disorder: A systematic review
Source: Alcohol Clin Exp Res (Hoboken). 2024 Nov 21;49(1):81–91. doi: 10.1111/acer.15492 (PMC11740172; doi:10.1111/acer.15492)
Supplement: Supplementary file 1 — Table S1. [file ACER-49-81-s001.docx]

Supplementary Table 1: Citations for the Diagnostic Criteria and Categorical Diagnoses Used in This Study.

| **Diagnostic System** | **Diagnostic Terms Used** | **Primary References** |
| --- | --- | --- |
| 4-Digit Code | FAS  pFAS  static encephalopathy, alcohol exposed  neurobehavioral disorder,  alcohol exposed | Astley, 2004  Astley, 2013 |
| Institute of Medicine (IOM) | FAS  pFAS  ARND | Hoyme et al., 2016 |
| Canadian | FAS  pFAS  ARND | Cook et al., 2016 |
| Australian | FASD with Three Sentinel Features  FASD with less than Three Sentinel Features | Brower et al., 2017 |
| DSM-5 ND-ND-PAE | ND-PAE | Kable, J and Coles CD., 2015  APA Diagnostic Manual, 2013 |
| Emory | FAS  pFAS  ARND | Coles et al., 2016 |
| Fetal Alcohol Syndrome Diagnostic Checklist (FASDC) | FAS  Other-FASD | Burd et al., 2010 |

Abbreviations: FAS=fetal alcohol syndrome; pFASD=partial fetal alcohol spectrum disorder; ARND=alcohol related neurodevelopmental disorder; FASD=fetal alcohol spectrum disorder; ND-PAE=neurodevelopmental disorder-prenatal alcohol exposure

**References**

Astley, S. J. (2004). Fetal alcohol syndrome prevention in Washington State: evidence of success. Paediatric and perinatal epidemiology, 18(5), 344-351. doi:10.1111/j.1365-3016.2004.00582.x [doi]

Astley SJ. Validation of the fetal alcohol spectrum disorder (FASD) 4-Digit Diagnostic Code. *J Popul Ther Clin Pharmacol.* 2013;20(3):e416-467.

Hoyme, H. E., Kalberg, W. O., Elliott, A. J., Blankenship, J., Buckley, D., Marais, A. S., . . . May, P. A. (2016). Updated Clinical Guidelines for Diagnosing Fetal Alcohol Spectrum Disorders. Pediatrics, 138(2), 4256. Epub 2016 Jul 4227. doi:10.1542/peds.2015-4256 [doi]

Cook, J. L., Green, C. R., Lilley, C. M., Anderson, S. M., Baldwin, M. E., Chudley, A. E., . . . Canada Fetal Alcohol Spectrum Disorder Research, N. (2016). Fetal alcohol spectrum disorder: A guideline for diagnosis across the lifespan. CMAJ : Canadian Medical Association journal = journal de l'Association medicale canadienne, 188(3), 191-197. doi:10.1503/cmaj.141593 [doi]

Brower, C., et al Australian guide to the diagnosis of fetal alcohol spectrum disorder. J Pediatr Health 2017, 53: 1021-1023

Kable, J. A., & Coles, C. D. (2015). Empirical evidence supporting the internal validity of the ND-PAE diagnosis. Alcohol and Clinical Experimental Research, 39, 226A.

American Psychiatric Association. (2013). Diagnostic and Statistical Manual of Mental Disorders (5th ed ed.). Washington, D.C.. American Psychiatric Association.

Coles, C. D., Gailey, A. R., Mulle, J. G., Kable, J. A., Lynch, M. E., & Jones, K. L. (2016). A Comparison Among 5 Methods for the Clinical Diagnosis of Fetal Alcohol Spectrum Disorders. Alcohol Clin Exp Res, 40(5), 1000-1009. doi:10.1111/acer.13032

Burd L, Klug MG, Li Q, Kerbeshian J, Martsolf JT. Diagnosis of fetal alcohol spectrum disorders: a validity study of the fetal alcohol syndrome checklist. *Alcohol.* 2010;44(7-8):605-614.
